# Violations of Hyperscaling in Phase Transitions and Critical Phenomena—In Memory of Prof. Ralph Kenna

**DOI:** 10.3390/e27080810

**Published:** 2025-07-29

**Authors:** Bertrand Berche, Yurij Holovatch

**Affiliations:** 1 Laboratoire de Physique et Chimie Théoriques, Université de Lorraine, BP 70239, CEDEX, 54506 Vandœuvre-les-Nancy, France; 2 𝕃^4^ Collaboration & Doctoral College for the Statistical Physics of Complex Systems, Leipzig-Lorraine-Lviv-Coventry, 79011 Lviv, Ukraine; 3 Yukhnovskii Institute for Condensed Matter Physics, National Academy of Sciences of Ukraine, 79011 Lviv, Ukraine; 4 Centre for Fluid and Complex Systems, Coventry University, Coventry CV1 5FB, UK; 5 Complexity Science Hub, 1030 Vienna, Austria


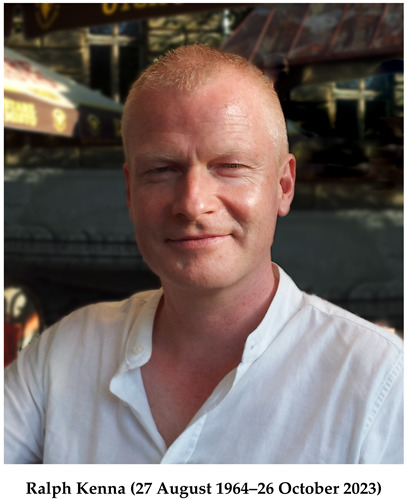


This Special Issue was initiated by Prof. Ralph Kenna, who at the time was a member of the Entropy Editorial Board. The chosen topic lay at the heart of his scientific endeavors, and we are deeply indebted to him for his significant contributions to this field. At the suggestion of the Entropy Editorial Board, we undertook the task of bringing this project to completion. We have done our utmost to honor Ralph’s vision, and we respectfully dedicate this Special Issue to his memory.

Ralph Kenna was born in Athlone (Ireland). He was a theoretical physicist and had very diverse centres of interest, such as statistical physics, complex systems, and Irish mythology. After a B.A. in Theoretical Physics (1985) and an M.Sc. (1988) from Trinity College Dublin, he completed his PhD at the University of Graz under Professor Christian Lang in 1993. Ralph then moved to the University of Liverpool from 1994 to 1997 and to Trinity College Dublin from 1997 to 1999. In 2002, he was hired at Coventry University, where he founded the Applied Mathematics Research Centre, joining, in 2018, the Centre for Fluid and Complex Systems. In 2016, he co-founded the $L^{4}$ Collaboration and Doctoral College for the Statistical Physics of Complex Systems, joining the Universities of Coventry, Leipzig, Lorraine, and the Institute for Condensed Matter Physics of the National Academy of Sciences of Ukraine in Lviv (ICMP). He was a Fellow of the Institute of Mathematics and its Applications, Member of the Institute of Physics, and an Advisory Board Member of the Middle European Cooperation on Statistical Physics. In 2019, for his important scientific contributions as well as for his initiative in different forms of collaboration with Ukraine and his engagement in the preparation of young scientists, he was conferred the title of Doctor Honoris Causa of the ICMP. Ralph Kenna passed away on 26 October 2023, and is buried in the St. Kieran’s Cemetery at Coosan, close to his home town, Athlone. Below, we will mention only some of his scientific results. A more complete description of his life and work can be found in [1].

In statistical physics, Ralph Kenna was a renowned expert in the study of critical phenomena and phase transitions via the analysis of the zeros of the partition function, an approach introduced by Lee and Yang in the 1950s [2,3] and further elaborated by Fisher in 1960s [4].

Together with his collaborators, Ralph Kenna was renowned for developing scaling relations involving logarithmic corrections [5,6], particularly at the upper critical dimension, then extending this to higher dimensions in 2012 [7], following the pioneering work of Michael Fisher on dangerous irrelevant variables [8,9]. This led to the introduction of the new pseudo-critical exponent (koppa) and its logarithmic counterpart (koppa-hat) to govern the finite-size scaling (FSS) of the correlation length and a new form for FSS, called QFSS, to replace standard prescription above the upper critical dimension [10,11,12,13,14]. Spin systems on scale-free networks also display logarithmic corrections that obey the scaling relations developed by Kenna [15].

Ralph Kenna has always been attracted by multidisciplinary subjects and has not hesitated during his career to tackle societal or human sciences problems. In 2010, Kenna and Berche quantified the notion of critical mass of academic research groups [16,17]. Using data from the UK’s Research Assessment Exercise 2008 and the French counterpart (then AERES), they tracked how research group quality depends on the size of the group. They found that quality rises linearly with group size up to a point, which they later identified as akin to the Dunbar number in anthropology [18]. Subsequently, together with his collaborators, he used scientometrics to predict the outcome of the UK’s Research Excellence Framework 2014. They established that correlations between metrics and peer review are poor [19].

In the field of comparative mythology, Ralph Kenna was a pioneer in applying complex network analysis to the study of Irish and other mythological traditions. His first paper on the topic [20] was downloaded over 30,000 times in 10 years and resulted in considerable media coverage in the international press. Other major works include investigations into the Sagas of Icelanders [21]. Kenna’s team found that whether the sagas are historically accurate or not, the properties of the social worlds they record are similar to those of real social networks. The Viking Age in Ireland as portrayed in Cogadh Gaedhel re Gallaibh was next tackled by his group [22]. They developed a measure to place hostility on a spectrum between civil war and international conflict. Their findings quantified and supported the traditional view of the Viking age in Ireland as one of international conflict and challenged recent revisionist claims. Kenna and co-workers also studied Ukrainian mythology. They compared the Kyiv bylyny cycle to other prominent European epics [23].

In the last months of his life, Ralph Kenna struggled with a serious, incurable illness. Continuing his scientific work was also part of this struggle. Among his various projects was the preparation of a Special Issue of the journal *Entropy*. Below is a brief description of the Special Issue scope written by Prof. Kenna:

*Universality is an emergent phenomenon, at least partially explained by the renormalization group. Because of universality, simplified theoretical models can deliver critical behaviour of real complex systems by trimming back to essentials such as dimensionality, symmetry group, and range of interaction. Universality classes of theoretical models and real systems are characterised by critical exponents that are linked through scaling relations between them. The scaling relations that involve dimensionality are referred to as hyperscaling. Due to the success of mean-field theory in highly connected systems, irrespective of the dimensionality of the systems, dimension-dependent hyperscaling is often said to fail there. That tenet was challenged recently with the introduction of new insights to the renormalization group aimed to rescue hyperscaling in high dimensions.*

*This Special Issue focuses on high-dimensional and other highly connected systems where hyperscaling is traditionally said to fail. We are interested in robustly supported explorations of how hyperscaling can or cannot be re-instated. The intent of this Special Issue is to capture “state of the art” research in high dimensions and high connectivity and as such we welcome new results and reviews of the highest standard. We are also interested in interdisciplinary applications.*

In the process of working on this Special Issue, its scope has been somewhat expanded. The articles offered to readers now contain, along with issues related to scaling above the upper critical dimensionality, other problems of the theory of phase transitions and critical phenomena [24,25,26,27,28,29,30,31,32]. We consider such an expansion of the subject appropriate, because the consideration of such problems places the question of hyperscaling violation and the peculiarities of scaling above the upper critical dimensionality in a broader context. Moreover, such an expansion of the subject enabled some of the authors who were closely associated with Ralph in their activities to participate in this Special Issue.

The first group of contributions addresses the general topic of finite-size scaling at or above the upper critical dimension—an area now commonly referred to as Q-finite-size scaling—a term coined by Ralph himself.

The opening article is a masterful review by Adelhardt, Koziol, Langheld, and Schmidt [24]. Long-range interactions play a crucial role in a wide variety of quantum systems, yet they remain notoriously challenging to handle from a theoretical standpoint. In this work, the authors provide a comprehensive overview of recent developments in the study of quantum magnets with long-range interactions. They discuss in detail the application of perturbative continuous unitary transformations and stochastic series expansion quantum Monte Carlo techniques to large finite systems. Finite-size scaling methods are then employed to extract the physical properties of the corresponding infinite systems. The review presents a thorough summary of the resulting quantum-critical behavior, including critical exponents across various models, and explores how long-range interactions can be tuned to explore quantum phase transitions beyond the upper critical dimension.

The second contribution is from Moueddene, Donoso, and Berche [25], and it pays tribute to Ralph’s favorite area of research, since this paper revisits the scaling relations among the so-called hatted critical exponents, originally introduced by Ralph Kenna, Des Johnston, and Wolfhard Janke. The authors propose alternative derivations for several of these relations. Notably, in the case of the scaling relation involving the behavior of the correlation function, they suggest a corrected form, having identified what they believe to be an error in the original formulation by Kenna and his collaborators.

The contribution by Young [26] deals with finite-size scaling above the upper critical dimension and, in particular, considers boundary-condition-dependent effects that arise there. The case with free-boundary conditions was investigated in detail by the author and is also discussed here, as it is still subject to current—possibly conflicting—investigations. Some numerical results are presented in addition to theoretical arguments.

Next, we turn to contributions in this volume that, while not related to Q-scaling, address broader issues in statistical mechanics. The paper by Folk and Holovatch [27] is dedicated to Ralph Kenna’s memory, but for technical reasons, it was not included in the online publication of the Special issue. In this article, the authors report on a part of the work on a larger project, also carried out with Ralph’s participation, which consisted of preparing the bilingual commented publication of Ernst Ising’s doctoral thesis [33]. The project has been completed this year [34]. Today, the Ising model stands as an archetype for describing collective ordering phenomena, renowned both within physics and across disciplines. Less widely known, however, is that Ernst Ising’s doctoral thesis—defended a century ago in 1924—contained more than the solution to what we now call the classical one-dimensional Ising model. Several additional problems, as well as the methods employed to solve them, are examined in this historical note. In particular, the authors highlight the combinatorial technique Ising used to compute the partition function of a chain of elementary magnets. In the thermodynamic limit, this approach yields a partition function expressed in terms of the roots of a specific polynomial. Remarkably, these “Ising roots” obtained via combinatorics coincide with the eigenvalues of the transfer matrix—a formalism only introduced later. The paper also discusses a three-state generalization of the two-state model proposed in Ising’s thesis but omitted from his well-known 1925 publication [35]. This extended model may be viewed as a precursor to the now-prevalent class of models featuring multicomponent order parameters.

Oliveira, Alves, Alves, Lima, and Plascak [28] investigate the Biswas–Chatterjee–Sen model of opinion dynamics on three-dimensional Solomon networks using extensive Monte Carlo simulations. Employing finite-size scaling analysis, they provide clear evidence that the model exhibits a second-order phase transition. The authors determine the critical exponents associated with the order parameter, its associated susceptibility, and the correlation length. Their results demonstrate that the model in three dimensions belongs to a universality class distinct from those found in the same model on one- and two-dimensional Solomon networks, as well as from the conventional Ising model defined on these networks.

In their contribution, Iglói and Lin [29] employ a real-space block renormalization group approach to investigate the critical behavior of the random transverse-field Ising spin chain with multispin interactions. They first recover the well-established results for the standard model with nearest-neighbor (two-spin) couplings. Extending their analysis to the case with three-spin interactions, they determine the location of the critical point and show that the associated phase transition is governed by an infinite-disorder fixed point. Notably, the typical correlation–length critical exponent appears to differ from that of the conventional random transverse-field Ising chain, indicating that the model with multispin couplings belongs to a distinct infinite-disorder universality class.

Gessert, Weigel, and Janke [30] analyze the zeros of the partition function in the complex temperature plane (Fisher zeros) and in the complex external field plane (Lee–Yang zeros) for a frustrated Ising model on the honeycomb lattice, featuring competing nearest-neighbor ($J_{1}>0$) and next-nearest-neighbor ($J_{2}<0$) interactions. They investigate the finite-size scaling behavior of the leading zeros and compare their findings with results from a more traditional scaling analysis, based on the logarithmic derivative of the magnetization and the magnetic susceptibility. Although both approaches are subject to significant corrections to scaling due to frustration, the nature of these corrections differs markedly, especially as the interaction ratio $R=J_{2}/J_{1}$ is varied. The analysis of the partition function zeros thus emerges as a valuable complement to conventional FSS methods. In particular, the scaling behavior of the zeros provides compelling evidence that the system remains within the Ising universality class down to R=−0.22, a regime where conclusions based on traditional FSS become less definitive. This demonstrates the utility of the zeros approach as a powerful additional tool for charting the phase diagrams of models strongly affected by corrections to scaling.

Henkel [31] investigates out-of-equilibrium dynamics in the one-dimensional Glauber–Ising model following a quench to zero temperature. Focusing on single-time and two-time correlation functions on a periodic chain of finite length *N*, he employs an analytical continuation method. In addition to providing further confirmation of finite-size scaling in non-equilibrium settings, this approach enables a detailed examination of the scaling behavior of the plateau height $C_{\infty }^{\left(2\right)}$, the asymptotic value approached by the two-time autocorrelator deep in the finite-size regime.

Burda and Johnston [32] exploit the correspondence between the partition function of an adsorbing Dyck walk model and the normalization of the Asymmetric Simple Exclusion Process (ASEP) to determine the thermodynamic limit of the locus of ASEP normalization zeros via a conformal mapping approach. They further discuss the equivalence of this method with an electrostatic analogy, which can also be used to derive the zero locus—both for the ASEP and for the related random allocation model. This dual perspective highlights the versatility and robustness of analytical techniques in determining the singularity structure of nonequilibrium systems.

With this collection of articles, their authors, together with the editors of the *Entropy* journal, express their deep respect for the memory of Ralph Kenna—an outstanding physicist and a wonderful person. Having conceived such a collection, he was unable to take part in its editing himself. However, the articles that you will read serve as a continuation of his ideas, and we believe that there will be many more such works.
